# Known data on the effectiveness of silver nano particles on root canal disinfection

**DOI:** 10.6026/97320630017218

**Published:** 2021-01-31

**Authors:** Iffat Nasim, V Vishnupriya, Zohra Jabin, Senthil Nathan

**Affiliations:** 1 Saveetha Dental College, Saveetha Institute of Medical and Technical Sciences, Saveetha University, Chennai, India; 2 Divya Jyoti College of Dental Sciences & Research, Modinagar, Distt.- Ghaziabad, U.P. India

**Keywords:** Antimicrobial colony forming units, disinfection root canal, silver nanoparticles, systematic review

## Abstract

The goal of endodontic treatment is the debridement and removal of the microbial ecosystem associated with the disease process. The need for root canal disinfectants increases especially in those cases where infection is resistant to the regular treatment and the outcome of endodontic therapy is often compromised. Therefore, it is of interest to document the known effectiveness of silver nanoparticle based root canal disinfectants with other root canal disinfectants on microbial load reduction during root canal disinfection. Known data shows that the overall risk of bias for the selected studies was moderate. Silver nanoparticle based root canal disinfectants showed superior reduction of microbial counts in majority of the studies. This data is limited to vitro studies with no clinical information to validate the use of antimicrobial properties of silver nanoparticles used as root canal disinfectant.

## Background

One of the objectives of a successful root canal treatment is to eliminate or reduce the presence of intra‐canal bacteria. An infected root canal system is a unique niche for the selective species of microorganisms [1]. It has been clearly defined that there is a microbial difference between primary endodontic treatment and retreatment [2]. Apical periodontitis, which persists after root canal treatment, possesses more complex etiological and therapeutic situation [3]. Certain species of microorganisms, especially Gram-positive facultative, possess greater resistance to antimicrobial agents used during endodontic treatment than anaerobes. Another important factor, which has become evident during the last few years, is that microbes in the root canals can grow not only as planktonic cells or in aggregates, co-aggregates, but they can also form biofilms consisting of a complex network of different microorganisms [4,5]. Biofilms are composed of micro colonies of bacterial cells that are distributed in a matrix which consists of exopolysaccharides, cell material etc in an aqueous solution. Bacterial biofilms are reported to be the most common cause of persistent inflammation [6]. As the morphology of root canal systems is complex it favours the growth of bacteria in the form of biofilms [7]. Numerous measures have been advocated to reduce the numbers of root canal microorganisms, including the use of various instrumentation techniques, irrigation regimens, and intracanal medications [8]. The chemo mechanical preparation of the root canal reduces endodontic infection. However, microorganisms are able to survive within the complex anatomy of the root canal system. In the field of endodontics, nanomaterials have been developed which focus to improve antimicrobial efficacy of root canal disinfectants, mechanical integrity of previously diseased dentin matrix, and tissue regeneration. Silver ions and salts are known for their wide antimicrobial effect [9]. They have been used since years in different fields in medicine, including wound dressings, catheters, and prostheses. [10-12]. AgNPs have applications in several areas of dentistry as endodontics, dental prostheses, implantology and restorative dentistry [13-16]. Because of their small size, they possess chemical, physical, and biological properties distinctive from those presented by traditional bulk materials [17]. Their smaller particles and large surface area provide potent antibacterial effects at a low filler level [18]. Other advantage provided by the small size is the possibility of silver nanoparticles to penetrate through cell membranes more readily, resulting in higher antimicrobial activity, [19] which is especially important since microorganisms in biofilms are more resistant to antimicrobial agents than planktonic pathogens [20]. Therefore, it is of interest to document the known effectiveness of silver nanoparticle based root canal disinfectants with other root canal disinfectants on microbial load reduction during root canal disinfection.

## Materials and methods:

### Protocol and registration:

A detailed protocol was developed for this systematic review in which the analysis and eligibility criteria were stated and documented, according to the Preferred Reporting Items for Systematic Reviews and Meta-Analyses PRISMA guidelines and The Cochrane Collaboration [21] and was registered with Open Science Framework [OSF][osf.io].

### Search Strategy:

The electronic search of the literature was conducted individually by two examiners [IN and ZJ] on the 'PubMed, Web of Science, EMBASE and Google Scholar' databases with the following MESH-terms with their synonyms and different combinations: Infected root canals AND Silver nanoparticles AND commonly used disinfectants AND antimicrobial effect. In addition, the reference lists of each paper containing as data were scanned to identify additional documents on the issues that had been missed. Only papers published in English were used. The electronic searches were conducted in July 2020. No restrictions on publication date were imposed.

### Population Intervention Control Outcome Question:

To address the aim of this systematic review, the following question was constructed based on the Population Intervention Control Outcome PICO principle: "Is antimicrobial efficiency of silver nanoparticles is better than the other antimicrobial agents used for root canal disinfection?".

###  Eligibility criteria:

Data extraction relied on the antimicrobial effect of Silver Nanoparticles in root canal infections. To further refine the search, the following inclusion criteria were adopted: Studies assessing antimicrobial activity of silver nanoparticles, report of outcomes of reduction in microbial load. Studies were excluded if they were animal studies or did not quantify the antimicrobial effect of silver nanoparticles or assessed the general activity of antimicrobial nanoparticles against microbial species non relevant to root canal infection or assessed the antimicrobial behavior of nanoparticles with no potential application in dental root canal or Reviews, book chapters and editorials with no experimental studies. 

### Our PICOS criteria were constructed as listed below:

#### Population: 

Teeth indicated for Root Canal/Inoculated root canals of extracted teeth with relevant microbial species /standard inoculums of relevant microbial species.

#### Intervention:

Exposure of the samples to Silver Nanoparticles with antimicrobial activity in root canal infections.

#### Comparison:

Treatment with commonly used root canal irrigants and/or intracanal medicaments.

#### Outcome:

Eradication of microbes or persistence in the acceptable concentration level.

#### Study design [S]:

In vivo studies, in vitro studies, ex vivo studies or clinical trials.

### Identification of Studies:

Two authors [IN and ZJ] independently reviewed all the selected studies by reading the titles and abstracts.

### Dataextraction:

Two authors [IN and ZJ] thoroughly studied all the included studies and independently collected the data.

### Qualityassessment 

Two authors [IN and ZJ] independently assessed the risk of bias. The quality assessment method was adopted from the methods used in previous systematic reviews [22,23].

The parameter was judged as low/high/unclear risk of bias. In case of unclear risk of bias, the authors were contacted through mail and doubts were cleared. Any disagreement between two authors was discussed with third author VP and problem was resolved. The parameter with high risk was marked as negative symbol with red color code. Low risk was marked as positive symbol and green color code. The studies were considered as low risk of bias if only one parameter had negative symbol and the studies having two or more negative symbols were considered to have moderate risk of bias.

## Results:

**Figure 1:**
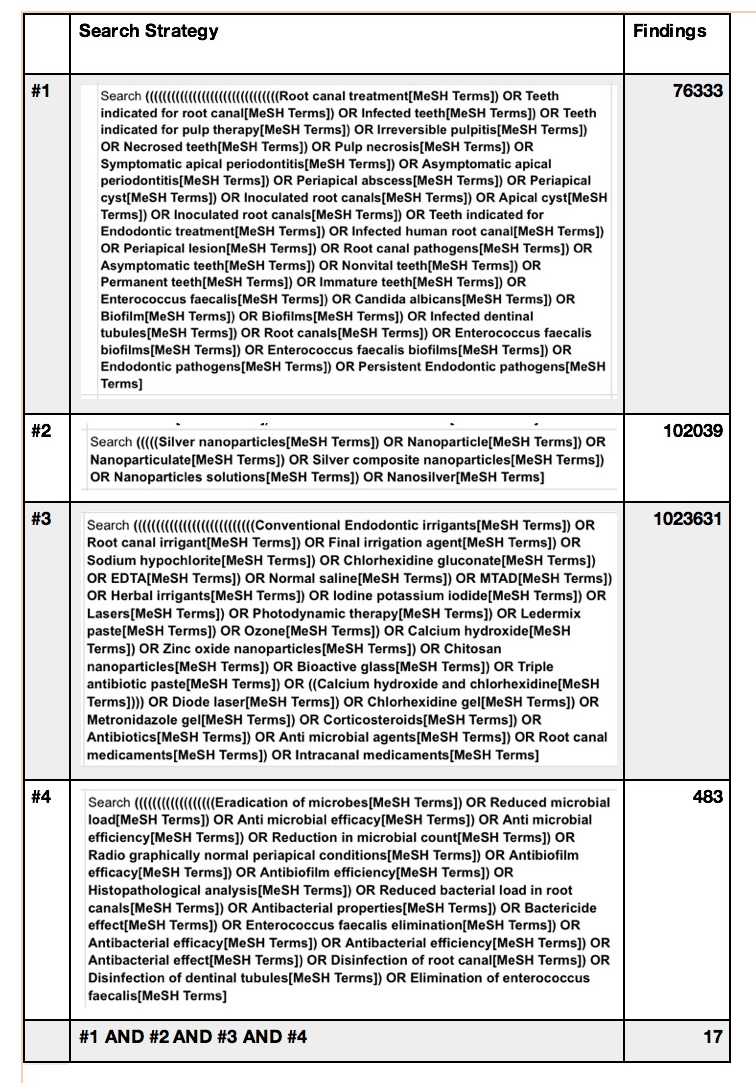
PubMed search strategy.

**Figure 2:**
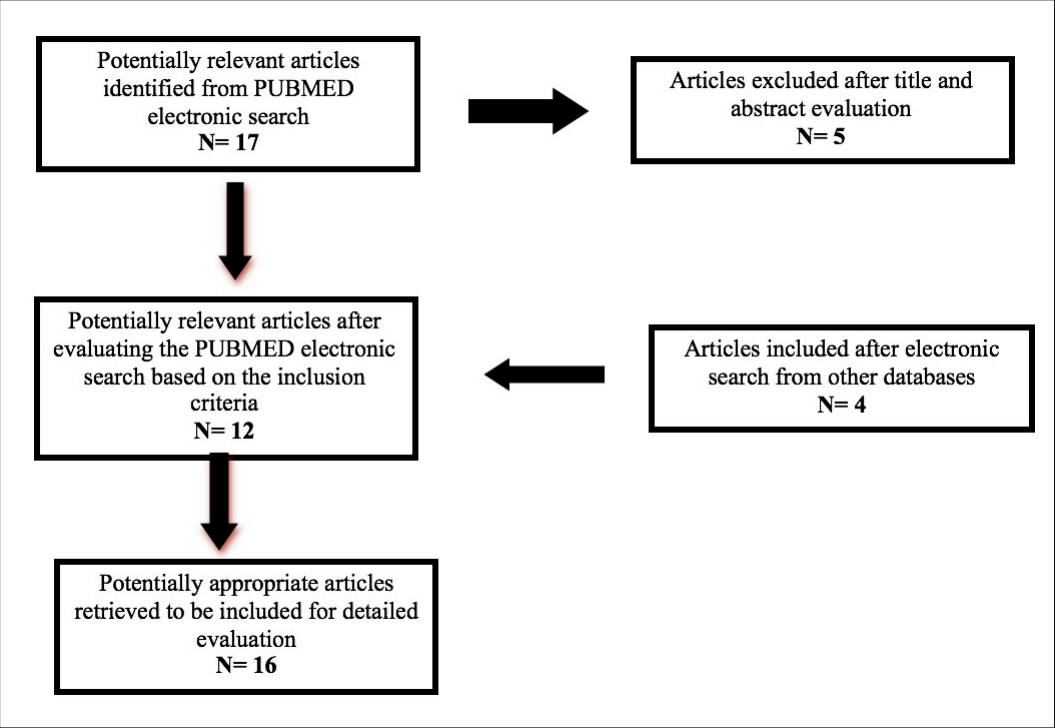
Search Flow Chart

**Figure 3:**
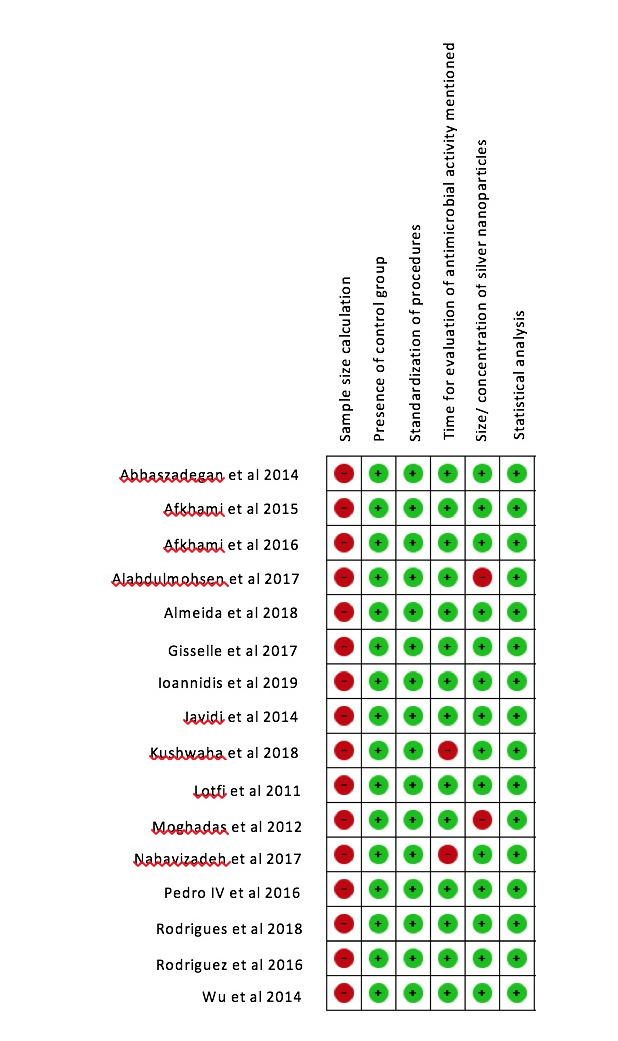
Risk of bias results of included studies [+] indicates low risk of bias, [-] indicates high risk of bias.

A total of seventeen titles and abstracts were identified after an electronic search in PubMed electronic database using the specific combination of terms and key words (Figure 1). Out of seventeen studies five studies were excluded, as they did not meet inclusion criteria. Reasons for exclusion was, in four studies Silver nanoparticles were not used, in one study comparison with other root canal disinfectants were not done. So potentially twelve articles were relevant from PubMed search. After search from other sources four articles were found. So total sixteen studies, which fulfilled the inclusion criteria, were included in this systematic review. (Figure 2) No clinical reports concerning the application of antimicrobial silver nanoparticles in endodontics was found. Thus the review was restricted to in vitro studies. The small number of studies and the heterogeneity among the studies such as difference in sample sizes and inclusion criteria among the included studies did not allow us to conduct a meta-analysis. The detailed data was collected from the selected studies. Table 1 (see PDF) gives the characteristics of the included studies. The risk of bias is summarized in Figure 3. Out of sixteen included studies twelve studies had low risk of bias and four studies had moderate risk of bias [24-27].

## Discussion:

Sixteen articles were selected for descriptive analysis. Till date there are no clinical trials done which have checked the efficacy of silver nanoparticles when used as root canal disinfectant either in the form of root canal irrigants or intracanal medicament. So in the present systematic review we considered only in vitro studies. On the basis of evidence extracted from the scientific literature, it is clear that silver NPs have unique properties allowing it to be one of the most commonly used metal NPs in dental application. Some studies favor the use of silver nanoparticles as root canal disinfectant but some studies gave contradictory findings too. Majority of the studies showed silver nanoparticles have comparable antimicrobial properties as gold standard sodium hypochlorite. Abbaszadegan et al compared antimicrobial activity of positively charged, negatively charged, neutral surface charged silver nanoparticles, 2.5 % NaOCl and 0.2% CHX.They found that positively charged silver nanoparticles were comparable to 2.5% NaOCl in eliminating E Faecalis. [28] Nano silver at low concentration had comparable bactericidal effect equivalent to 2.25% or 5.25% NaOCl. [26,27, 29,30] Almeida et al reported that Silver nanoparticles showed similar antimicrobial activity compared to 5% NaOCl, 2% Chlorhexidine. [31] It is an important point to note that when silver nanoparticles were combined with commonly used root canal disinfectants they were able to exert better antimicrobial activity. Afkhami et al used silver nanoparticles as a vehicle for calcium hydroxide and found that the antimicrobial activity was better when compared to other vehicles but this effect was noticed for short term. The antimicrobial activity seems to be comparable with other combinations when tested again after one month. [32] Giselle et al combined Silver nanoparticles with other root canal irrigants and they found that the combined irrigants were able to achieve complete bacterial elimination but they were not proved to be better than sodium hypochlorite. They also noted that there was increase in bacterial count with all the tested root canal irrigants after 7 days. [33] Javidi et al tested the antimicrobial activity of intracanal medicaments after one day and seven days.The combination of nano silver and calcium hydroxide showed better antimicrobial activity when compared to calcium hydroxide used alone. The combination of silver nanoparticles and calcium hydroxide intracanal medicament was able to exert antimicrobial activity after one-day exposure and remained unchanged after 7 days [34]. Afkhami et al found that silver nanoparticles were equivalent to 2.5% NaOCl in reducing the microbial load and antimicrobial activity was increased when silver nanoparticles were combined with diode laser and photodynamic therapy [35]. Kushwaha et al combined Silver nanoparticles with laser and observed excellent antimicrobial properties [25]. According to Ioannidis et al silver nanoparticle graphene oxide combination had greater antimicrobial activity when compared to 17% EDTA, 2% CHX and 1% NaOCl but showed lesser antimicrobial activity when compared to 2.5% NaOCl [36]. An important noteworthy finding was shown by Wu et al. They evaluated the anti microbial efficacy of silver nanoparticles in the form of root canal irrigants and intracanal medicaments and found that antimicrobial activity is dependent on mode of application and silver nanoparticles when used as intracanal medicaments have better antimicrobial efficacy when compared to root canal irrigants [37]. The present systematic review also found some contradictory results in which Silver nanoparticles were not found better than sodium hypochlorite. [24,38,39] Majority of the included studies in this review showed an enhanced effect of silver nano particulate systems to combat dental root canal infections. After reviewing all the studies and according to the evidence available silver nanoparticles can be considered as an adjunct to existing root canal disinfectants. The future of these promising approaches lies in the development of better techniques for preparing efficient antimicrobial nanoparticles in addition to the highest safety for patients and to assess their toxic effects in clinical situations.
